# COS-Speech: protocol to develop a core outcome set for dysarthria after stroke for use in clinical practice and research

**DOI:** 10.1186/s13063-022-06958-7

**Published:** 2023-01-25

**Authors:** C. Mitchell, A. Bowen, P. Conroy, B. M. Whelan, S. J. Wallace, A. Dancer, K. Woodward-Nutt, J. J. Kirkham

**Affiliations:** 1 School of Health Sciences, Faculty of Biology, Medicine and Health, University of Manchester, Manchester, England; 2 Geoffrey Jefferson Brain Research Centre, The Manchester Academic Health Science Centre, Northern Care Alliance and the University of Manchester, Manchester, UK; 3 School of Health and Rehabilitation Sciences, The University of Queensland, Brisbane, Australia; 4 COS-Speech Healing, Empowered and Recovering from Dysarthria (HEARD) group, Manchester, UK; 5 Research and Innovation, Northern Care Alliance NHS Foundation Trust, Salford, UK; 6 Centre for Biostatistics, The University of Manchester, Manchester Academic Health Science Centre, Manchester, UK

**Keywords:** Stroke, Dysarthria, Core outcome set, Delphi process

## Abstract

**Background:**

Dysarthria after stroke is when speech intelligibility is impaired, and this occurs in half of all stroke survivors. Dysarthria often leads to social isolation, poor psychological well-being and can prevent return to work and social lives. Currently, a variety of outcome measures are used in clinical research and practice when monitoring recovery for people who have dysarthria. When research studies use different measures, it is impossible to compare results from trials and delays our understanding of effective clinical treatments. The aim of this study is to develop a core outcome set (COS) to agree what aspects of speech recovery should be measured for dysarthria after stroke (COS-Speech) in research and clinical practice.

**Methods:**

The COS-Speech study will include five steps: (1) development of a long list of possible outcome domains of speech that should be measured to guide the survey; (2) recruitment to the COS-Speech study of three key stakeholder groups in the UK and Australia: stroke survivors, communication researchers and speech and language therapists/pathologists; (3) two rounds of the Delphi survey process; (4) a consensus meeting to agree the speech outcomes to be measured and a follow-up consensus meeting to match existing instruments/measures (from parallel systematic review) to the agreed COS-Speech; (5) dissemination of COS-Speech.

**Discussion:**

There is currently no COS for dysarthria after stroke for research trials or clinical practice. The findings from this research study will be a minimum COS, for use in all dysarthria research studies and clinical practice looking at post-stroke recovery of speech. These findings will be widely disseminated using professional and patient networks, research and clinical forums as well as using a variety of academic papers, videos, accessible writing such as blogs and links on social media.

**Trial registration:**

COS-Speech is registered with the Core Outcome Measures in Effectiveness Trials (COMET) database, October 2021 https://www.comet-initiative.org/Studies/Details/1959.

In addition, “A systematic review of the psychometric properties and clinical utility of instruments measuring dysarthria after stroke” will inform the consensus meeting to match measures to COS-Speech. The protocol for the systematic reviews registered with the International Prospective Register of Systematic Reviews. PROSPERO registration number: CRD42022302998.

**Supplementary Information:**

The online version contains supplementary material available at 10.1186/s13063-022-06958-7.

## Background

Dysarthria is a type of communication impairment that commonly occurs after stroke affecting speech. It presents in a variety of ways, with varying degrees of severity, but usually leads to impaired and less intelligible speech [1]. We know that dysarthria affects 52% of stroke survivors, whilst 41% of stroke survivors have aphasia [2]. We also know that 28% have aphasia and dysarthria together compared to having only dysarthria (24%) or only aphasia (12%) [2]. Stroke survivors with dysarthria have poorer health outcomes, worse psychological well-being, social isolation and can prevent people from returning to their previous work and social lives, compared to those with no communication impairment, regardless of the severity of their dysarthria [3–7].

Intervention research in dysarthria is limited, a Cochrane review found five studies for inclusion [8]. This compares poorly to aphasia research which has 57 studies in this similar stroke population [9]. The findings from the dysarthria Cochrane review found despite the small number of trials, there were eleven different outcome measures used and at different time points, with only two studies using the same measure. This means it is difficult to compare findings from these trials, where combined data from small studies could give us clearer information, even more important with so few research studies. The creation of a core outcome set (COS) is urgently needed to improve the quality and efficiency of future research in dysarthria to reduce the widely recognised problem of waste in medical research [10].

A COS is a standard set of outcomes which should be measured and reported in all studies related to a particular health condition [11]. A COS allows research findings to be compared, combined, and contrasted and reduces reporting bias [12]. There are many examples of COS that have been developed and are in use across related stroke conditions that continue to contribute to clinical and research practices [13]. In 2019, a COS for aphasia (language impairment) after stroke was successfully developed [14]. There are challenges to implementation of COS [15–17] and uptake across different areas of health is variable. Some of the challenges related to the lack of appropriate measures, limited involvement of key stakeholders and a lack of awareness of the COS once developed [16].

The aim of our study is to develop a consensus-based COS for dysarthria after stroke to agree ‘what’ outcomes of speech to measure and ‘how’ best to measure them. We intend to recruit relevant key stakeholders, establish what measures are appropriate and ensure we disseminate this COS in both clinical and research communities [15]. A COS for dysarthria would improve consistent and relevant outcome reporting for people with dysarthria in both clinical and research settings.

### Scope

This COS-Speech relates to all adults who have dysarthria after stroke and applies to intervention research and assessment in clinical practice.

## Methods

This protocol, version 1, is written following the Core Outcome Set standards for development recommendations (COS-STAD) and the Core Outcome Set Standardised Protocol (COS-STAP) checklist [18, 19], completed checklist in Additional file 1.

### Study process

The COS-Speech development will progress through five steps: (1) generate the COS-speech long list of domains; (2) recruit to the COS-Speech; (3) carry out the two-round Delphi survey; (4) hold a consensus meeting to agree domains and a follow-up meeting to match existing instruments to the agreed outcome measures (systematic review of all measures being carried out in parallel with this study registered on PROSPERO registration number CRD42022302998 at https://www.crd.york.ac.uk/PROSPERO to inform step 4); (5) dissemination and implementation.

**Fig. 1:**
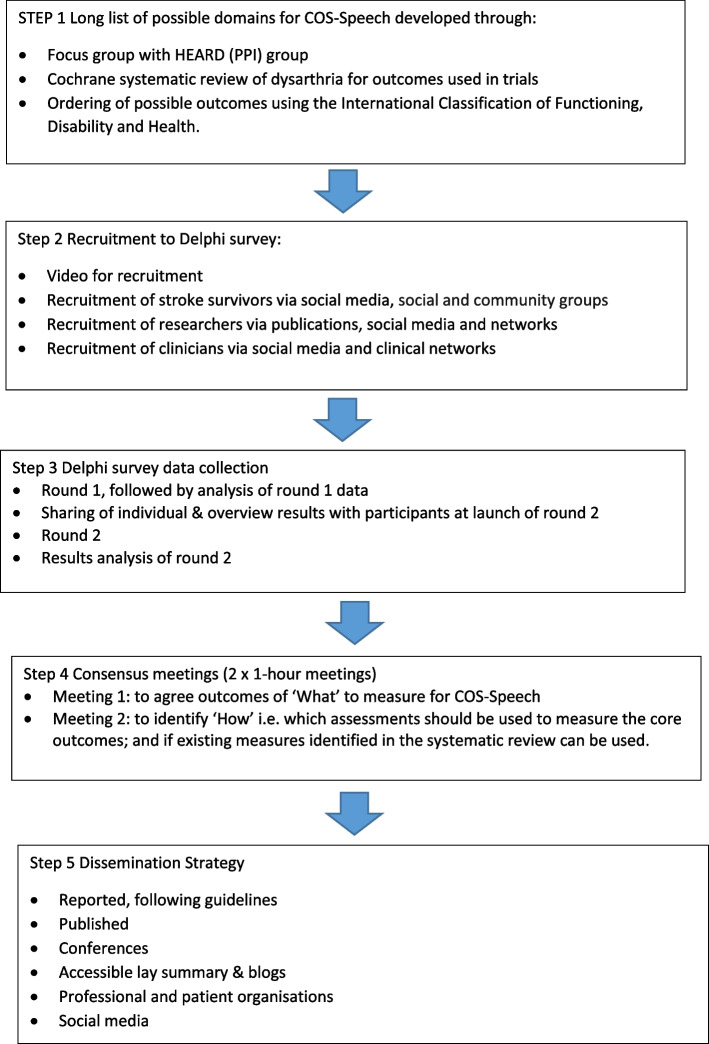
Workflow for COS-Speech study process

This approach follows the CS-COUSIN roadmap, a framework for developing core sets of outcome and measurements [20]. A summary of the method we will use is shown in a flow chart in Fig. 1.

### Step 1: COS-Speech outcome long list

A long list of possible outcome domains to include in COS-Speech will be developed. This initial long list of outcomes for inclusion in the consensus process will be generated from the list of outcomes routinely used in efficacy or effectiveness trials found in the dysarthria Cochrane review [8] (Additional file 2). The study team and HEARD group will generate other possible outcomes. This will be an iterative process. The HEARD group will meet and identify areas which they believe are important to measure; this list will be shared with the research team and additional areas added. HEARD group members will review and add to the list until they are satisfied with the list. The HEARD group will also assist in ensuring that the long list is written in accessible language and to provide plain language summaries of all the potential outcome domains to be considered. The long list will be grouped in relevant groups of body functions, activities and participation, major life areas, environmental factors, attitudes, body structures and personal factors using the international classification of functioning, disability and health (ICF) (WHO, 2007).

### Step 2: COS-Speech recruitment

This study will recruit from the three stakeholder groups with the following inclusion criteria: (a) stroke survivors with lived experience of dysarthria, (b) speech and language therapists/ pathologists with experience of treating patients with dysarthria, (c) communication researchers from any professional background. Participants will complete the survey online in their own homes, with support using the computer if needed. Recruitment will be limited to participants living in the United Kingdom or Australia due to the time involved in obtaining local ethics approval for each country involved.

#### Clinicians

We intend to recruit speech and language therapy clinicians from national clinical excellence networks for speech and language therapists/pathologists working in adult acquired conditions including Stroke Foundation and the Australian Aphasia Association in Australia and adult acquired clinical excellence networks across the UK, and via Twitter (@dysarthrialife).

#### Researchers

We will contact researchers through existing researcher networks such as the Collaboration for Aphasia Trialists, Queensland Aphasia Research Centre Clinical and Consumer Affiliates, Centre of Research Excellence in Aphasia Recovery and Rehabilitation; researchers identified through the Cochrane review of interventions for dysarthria and other published studies, and through Twitter.

#### Stroke survivors

We will seek to identify stroke survivors through stroke support groups and other community groups across the UK and Australia and via Twitter. Stroke survivors will be asked for some basic demographic data, age range, time post-stroke, male or female and ethnic background when they complete the survey.

A short video to explain the project (https://youtu.be/Axl_1MTYovQ) will also be made available on the study webpage (https://sites.manchester.ac.uk/cos-speech/) and on Twitter.

There is no agreed optimal number of people who should be involved in a Delphi study which can often vary widely [21]. We intend to recruit approximately 75 participants in total across the three stakeholder groups. Forty-five in the UK; 15 speech and language therapists, 15 communication researchers and 15 stroke survivors and 30 in Australia; 10 speech and language therapists, 10 communication researchers and 10 stroke survivors. Participants will be sent the participant information, confirm they have read this and give consent to continue to the electronic survey, all personal data will be stored securely and pseudonymised. The intention is to have at least 10 participants from each group complete the final round of the Delphi survey, this is consistent with other COS developments [22].

### Step 3: Delphi survey

Round 1 of the Delphi survey will be carried out using the outcomes generated in Step 1. Participants will rate each outcome on a scale of importance with 9 being the most important and 1 being the least important.

Rankings of 7–9 indicate critical importance, 4–6 outcomes that are important but not critical, whilst ratings of 1–3 are of limited importance using the Grading of Recommendations Assessment, Development and Evaluations (GRADE) scale [23]. Participants will be invited to put other outcomes forward that they think are important but had not been included in the survey. Following our analysis of round 1 data, we will consider what changes need to be made to any of the questions, based on the data and comments from participants completing round 1. This may involve including more outcomes or reducing other outcomes for example. In round 2 of the Delphi process, participants will see the results for all three stakeholder groups (the percentage distribution of scores) from round 1 and will be reminded of their own score from round 1. Participants will be alerted to any changes to the questions from round 1 to round 2 and encouraged to complete the full survey. Each round will be online via DelphiManager and open for three or four weeks and reminder emails will be sent after the initial contact. Attrition rates will be monitored, the survey could be kept open for longer, with more reminder emails to increase responses. The responses from the researchers, speech and language therapists and stroke survivor members involved in the e-Delphi survey will be analysed separately to assess differences in priority. In round 2, the response scoring options will be the same as the first round and include any new outcomes (if relevance agreed by the PPI and study management committee). Participants will be alerted where questions have been amended from feedback from round 1. All responses will be analysed anonymously, according to each stakeholder group and included in the analysis, even where people have not fully completed the survey. The questions are generated in random order, by each domain group, for each participant in order to reduce the impact of any missing data.

### Step 4.1: COS-Speech consensus meeting to agree domains

Following the two Delphi rounds, there will be an online meeting to consolidate COS-Speech from the Delphi results. All participants will be invited to attend the consensus meetings provided they completed both rounds, expressed an interest in attending the consensus meeting and can attend at a time that is convenient to the majority. Participants will need to give written consent to attend these meetings, which will be recorded on an encrypted device for data analysis purposes and then deleted. Using these criteria, we aim to have at least two participants from each stakeholder group attend the consensus meetings with representation from both the UK and Australia. To ensure ‘fair’ representation, participants may be purposively selected to ensure that similar numbers from each stakeholder group and UK/Australia mix are present at the meeting.

The meeting will be chaired by an independent facilitator. During the meeting, the results of the two-round Delphi will be presented according to the definition of consensus (Table 1). Where outcomes have reached ‘consensus in’ or ‘consensus out’ from the Delphi survey, participants will have the opportunity to voice opinions should they disagree with the inclusion/exclusion of the outcome in the COS. Participants who disagree will be invited to provide further information before voting will take place. All consensus meeting participants will then vote either ‘yes’ the outcome should be included in the COS or ‘no’ the outcome should not be included in the COS, with a percentage of 70% of all participants voting ‘yes’ required for the outcome to be included in the final COS. Where outcomes have not reached consensus during the Delphi, they will be discussed in more detail and participants of the consensus meeting will be invited to participate in the same yes/no vote. All outcomes retained will then be included in the final COS-Speech.

**Table 1:** Consensus definition for COS-Speech

**Consensus in** Consensus that the outcome should be included in the core outcome set	Uncertainty about the importance of the outcome	**Consensus out** Consensus that the outcome should not be included in the core outcome set.
70% or more participants in each stakeholder group scoring as 7–9 and fewer than 15% in each stakeholder group scoring as 1–3	Any other scoring	50% or fewer participants scoring it 7–9 in each stakeholder group

### Step 4.2: COS-Speech consensus meeting to agree on measurement instruments

Following the first consensus meeting, we will hold a further online consensus meeting to determine whether we can link the agreed outcomes in COS-Speech to existing measures. The measures will have been identified through a systematic review being completed in parallel to the study to identify existing measures and evaluate psychometric properties and clinical utility following the Outcome Measures in Rheumatology (OMERACT) guidance and COSMIN [24]. This systematic review will identify all available ways of measuring dysarthria after stroke, it will consider the population, participant numbers, validity and reliability of the instrument in question, clinical utility and grade this according to overall quality of the data. All participants attending the first meeting will be invited to this second meeting and we will seek consensus for the most popular measurement instrument (i.e. most number of votes) that could be used for each category of COS-Speech.

### Step 5: Dissemination process

We will adopt a multi-method approach to dissemination of COS-Speech. We will report the development of this COS-Speech according to the COS-STAR (Core Outcome Set-STandards for Reporting) guidelines [25]. The final COS-Speech and agreed measures will be published in an open-access journal. The findings will be summarised and published in the UK national speech and language therapy professional quarterly publication and the equivalent publication internationally. After publication COS-Speech will be available on the COMET database and has already been registered with COMET (https://www.comet-initiative.org/Studies/Details/1959).

Our intention is to present COS-Speech at national and international stroke research conferences to ensure researchers are aware of the study results. We will also present at professional (speech and language therapy/pathology) conferences in the UK and internationally. In addition, we will present at patient organisations or forums to ensure clinicians and patient groups of stroke survivors are aware of COS-Speech. We will present at relevant special interest groups and clinical excellence networks in the UK and internationally.

All participants will receive a copy of the report and/or access to a video of the findings. Our HEARD PPI group will contribute to lay summaries and blogs to explain the findings in accessible formats for other stroke survivors. Relevant content relating to COS-Speech will be shared on various social media platforms, including Twitter, Instagram and Facebook to inform the dysarthria online community about this research.

### Patient, public involvement

Annette Dancer (AD), the PCPI co-applicant/ grant holder, has worked as a member of the research team throughout the planning and application stages of the project. She will continue to do so throughout the study and has been involved in setting up a dedicated COS-Speech advisory group called ‘HEARD’ (Healing, Empowered and Recovering from Dysarthria). The HEARD group consist of 3 stroke survivors with first-hand experience of dysarthria. They are part of the research team and provide input and advice on all research activities from study documentation through to the dissemination of results. The group will meet, together with the Chief Investigator and other members of the research team, monthly during the course of the study as required.

AD represents the HEARD group on the study management committee and will feed information in and out of both groups. In her absence, another member of the HEARD group will represent her at the management committee. HEARD group members will also have access to other members of the research team should they wish to discuss issues outside of meetings.

HEARD group members will be offered an honorarium and expenses in line with INVOLVE best practice.

Ethical approval has been obtained to permit the HEARD group members to participate in the study. The University of Manchester, UK, Ethics committee 1 (REC1: 28/03/2022, ref: 2022-13303-22550).

### Study management committee

The study management committee includes the principal investigator (CM), joint lead investigator (JJK), co-applicants and collaborators from the UK (AB, PC, AD, KWN) and Australia, (SW, BMW). This includes researchers, methodologists and researchers with experience in COS development, clinicians with experience of both dysarthria and aphasia, and a stroke survivor representing the HEARD patient and public involvement group who have first-hand experience of living with dysarthria. Management Committee members who are communication researchers may choose to participate in the survey.

## Discussion

This protocol describes the methods that we will use to develop a COS for dysarthria after stroke (COS-Speech) using surveys to gain initial agreement and a consensus meeting to finalise the decisions. There is currently no published COS for speech recovery after stroke that can be used in clinical practice or research. We will adhere to the published standards expected in developing a core outcome after stroke and follow published guidance [19, 25]. It is essential that the outcomes for speech in people who have dysarthria after stroke are relevant to the individual. The involvement of people with lived experience in the development of this project and their involvement as participants means that clinical practice and research will reflect this. It will also be important to include the perspectives of those delivering clinical practice and research to ensure we consider all aspects of speech recovery. Developing a standardised COS for speech recovery after stroke will allow synthesis of future research, reduce waste of resources in research which will speed up the development of intervention approaches to dysarthria. We intend to produce a patient-focused, accessible, and comprehensive guide to the outcome set selection for dysarthria after stroke which will be sent to the participants, relevant patient communities and on social media. The intention is for this to be used in research internationally, although one limitation of this study is that we have only sought consensus from the UK and Australia. We will seek to disseminate this work to other countries. We intend for COS-Speech to be used in clinical practice and future research and will disseminate to these networks through presentation and publication.

We recognise and acknowledge the following study limitations:

- The scope of the project is limited to UK/Australia. We would very much have liked to have been able to recruit to all stakeholder groups from additional countries to have a broader ethnic and cultural mix. However, this was not possible as ethical approval would be needed from the country of residence for each participant. Opportunities to implement the COS for speech in other countries will be explored on completion of the study.
- The team recognise that the E-Delphi method may make participation in the study difficult for some stroke survivors. The research team will attempt to address this by offering support to complete the questionnaire- face to face where possible or remotely by phone/zoom, etc.

## Trial status

First round of Delphi July 2022, second round October 2022–November 2022, consensus meetings February and March 2023.

## Supplementary Information

**Additional file 1.** Core Outcome Set Standardised Protocol (COS-STAP) checklist.

**Additional file 2.** Outcomes used in trials from Cochrane review to guide survey.

## Abbreviations

COMET: Core Outcome Measures in Effectiveness Trials
COS: Core outcome set
COS-Speech: Core Outcome Set for dysarthria after stroke
COS-STAP: Core Outcome Set Standardised Protocol
COS-STAR: Core Outcome Set-Standards for Reporting guidelines
GCP: Good Clinical Practice
GDPR: General Data Protection Regulation
OMERACT: Outcome Measures in Rheumatology
CS-COUSIN: Cochrane Skin – Core Outcome Set Initiative
GRADE: Grading of Recommendations Assessment, Development and Evaluations
HEARD: Healing, Empowered and Recovering from Dysarthria
PROSPERO: Prospective Register of Systematic Reviews
