# Supplementary material for: COS-Speech: protocol to develop a core outcome set for dysarthria after stroke for use in clinical practice and research
Source: Trials. 2023 Jan 25;24:57. doi: 10.1186/s13063-022-06958-7 (PMC9878925; doi:10.1186/s13063-022-06958-7)
Supplement: Supplementary file 1 — Additional file 1. Core Outcome Set Standardised Protocol (COS-STAP) checklist. [file 13063_2022_6958_MOESM1_ESM.pdf]

Additional File 1

Core Outcome Set Standardised Protocol (COS-STAP) checklist

**Core Outcome Set-STandards Protocol Items: The COS-STAP Statement Checklist**

| SECTION/TOPIC | ITEM No. | CHECKLIST ITEM | REPORTED ON PAGE NUMBER |
|---|---|---|---|
| TITLE/ABSTRACT | | | |
| Title | 1a | Identify in the title that the paper describes the protocol for the planned development of a COS | 1 |
| Abstract | 1b | Provide a structured abstract | 2 and 3 |
| INTRODUCTION | | | |
| Background and objectives | 2a | Describe the background and explain the rationale for developing the COS, and identify the reasons why a COS is needed and the potential barriers to its implementation | 4 and 5 |
| | 2b | Describe the specific objectives with reference to developing a COS | 4 and 5 |
| Scope | 3a | Describe the health condition(s) and population(s) that will be covered by the COS | 5 |
| | 3b | Describe the intervention(s) that will be covered by the COS | 5 |
| | 3c | Describe the context of use for which the COS is to be applied | 5 |
| METHODS | | | |
| Stakeholders | 4 | Describe the stakeholder groups to be involved in the COS development process, the nature of and rationale for their involvement and also how the individuals will be identified; this should cover involvement both as members of the research team and as participants in the study | 5,6 and 8 |
| Information sources | 5a | Describe the information sources that will be used to identify the list of outcomes. Outline the methods or reference other protocols/papers | 8 |
| | 5b | Describe how outcomes may be dropped/combined, with reasons | 10 |
| Consensus process | 6 | Describe the plans for how the consensus process will be undertaken | 10 |
| Consensus definition | 7a | Describe the consensus definition | 10 and 11 |
| | 7b | Describe the procedure for determining how outcomes will be added/combined/dropped from consideration during the consensus process | 10 and 11 |
| ANALYSIS | | | |
| Outcome scoring/feedback | 8 | Describe how outcomes will be scored and summarised, describe how participants will receive feedback during the consensus process | 9 |
| Missing data | 9 | Describe how missing data will be handled during the consensus process | 10 |

| | | | |
|---|---|---|---|
| ETHICS and DISSEMINATION | | | |
| Ethics approval/informed consent | 10 | Describe any plans for obtaining research ethics committee/institutional review board approval in relation to the consensus process and describe how informed consent will be obtained (if relevant) | 14 |
| Dissemination | 11 | Describe any plans to communicate the results to study participants and COS users, inclusive of methods and timing of dissemination | 11 |
| ADMINISTRATIVE INFORMATION | | | |
| Funders | 12 | Describe sources of funding, role of funders | 14 |
| Conflicts of interest | 13 | Describe any potential conflicts of interest within the study team and how they will be managed | 13 |

*From: Kirkham JJ, Gorst S, Altman DG, et al. (2019) Core Outcome Set-STAndardised Protocol Items: the COS-STAP Statement. Trials 20, 116. https://doi.org/10.1186/s13063-019-3230-x*
