# Supplementary material for: COS-Speech: protocol to develop a core outcome set for dysarthria after stroke for use in clinical practice and research
Source: Trials. 2023 Jan 25;24:57. doi: 10.1186/s13063-022-06958-7 (PMC9878925; doi:10.1186/s13063-022-06958-7)
Supplement: Supplementary file 2 — Additional file 2. Outcomes used in trials from Cochrane review to guide survey. [file 13063_2022_6958_MOESM2_ESM.pdf]

Additional File 2: Outcomes used in trials from Cochrane review to guide survey

Impairment	Outcomes related to how speech and voice is affected
	Outcomes related to the specific muscles involved in speaking
	Outcomes relating to detailed speech and voice assessment
Activity	Outcomes related to conversations
	Outcomes related to everyday speaking
Participation	Outcomes related to everyday life activities
	Outcomes to psychological well-being
